# Childhood Trauma Is Nominally Associated With Elevated Cortisol Metabolism in Severe Mental Disorder

**DOI:** 10.3389/fpsyt.2020.00391

**Published:** 2020-05-14

**Authors:** Monica Aas, Torill Ueland, Amina Inova, Ingrid Melle, Ole A. Andreassen, Nils Eiel Steen

**Affiliations:** ^1^NORMENT, Psychosis Research Unit, Division of Mental Health and Addiction, Oslo University Hospital, Oslo, Norway; ^2^Department of Psychology, University of Oslo, Oslo, Norway; ^3^NORMENT, Institute of Clinical Medicine, University of Oslo, Oslo, Norway

**Keywords:** childhood trauma and adversity, cognitive function, clinical features, schizophrenia, bipolar disorders, cortisol metabolism

## Abstract

**Objective:**

Individuals exposed to childhood trauma display longstanding modifications of the Hypothalamic–Pituitary–Adrenal (HPA) axis, as well as cognitive impairments. Schizophrenia spectrum disorder (SZ) and bipolar disorders (BD) are characterised by higher prevalence of childhood trauma, abnormal HPA axis, and cognitive dysfunction. Elevated cortisol metabolism was recently demonstrated in both disorders. However, it is yet to be established if childhood adversity is associated with cortisol metabolism in this population, and how this may be associated with cognitive function.

**Methods:**

One-hundred-and-fourteen participants with a DSM-IV SZ or BD diagnosis took part in the study. Diagnoses were evaluated by the Structured Clinical Interview for DSM-IV Axis I disorders (SCID-I). Estimated cortisol metabolizing activity (5α-reductase and 5β-reductase) was assessed by urinary free cortisol, and metabolites. All patients underwent cognitive assessment and completed the Childhood Trauma Questionnaire.

**Results:**

Estimated 5β-reductase activity was elevated in participant with childhood physical abuse (r = 0.26, p = 0.005). After adjusting for age, sex and diagnosis, physical abuse was still nominally associated with elevated 5β-reductase. Moreover, only high 5α-reductase activity was negatively correlated with working memory and executive performance (r = −0.23, p = 0.01; r = −0.19, p = 0.05, respectively), however this disappeared after adjusting for age, sex and diagnosis. Cortisol metabolism did not mediate the association between childhood trauma and cognitive function.

**Conclusions:**

Our study indicates that childhood physical abuse is associated with elevated cortisol metabolism (5β-reductase) in adults with a SZ or BD disorder. However, our study did not support cortisol metabolism as a mediator between childhood trauma experiences and cognitive function within these disorders.

## Introduction

Individuals exposed to high levels of childhood trauma display long-standing modifications of the biological stress response system, the Hypothalamic–Pituitary–Adrenal (HPA) axis (1). Patients with a schizophrenia (SZ) or a bipolar disorder (BD) report more often childhood traumatic experiences than the general population (2, 3), with HPA axis correlates (1). Both animal (4, 5) and human (6, 7) studies support that early life stress can lead to long-lasting stress-sensitization and dysregulation of the biological stress response. These early life experiences are thought to contribute to the progress of psychosis in susceptible individuals (1). Childhood trauma victims have a heightened negative reaction to distressing experiences later in life (8), elevated cortisol levels over time (9) and blunted responses on stress reactivity tests (10). Both childhood trauma experiences and HPA axis abnormalities are associated with poorer cognitive function in psychotic disorders (1). Nevertheless, to date it is yet to be established if childhood adverse events are also associated with abnormalities in cortisol metabolism in patients with SZ or BD (11) or how this is related to cognitive and clinical features of the disorders.

Studies investigating cortisol metabolism have been performed in Post-Traumatic Stress Disorder (PTSD) (12–14). As discussed by (13), several stress related neuropsychiatric disorders, including PTSD, and chronic fatigue paradoxically exhibit somewhat low levels of cortisol (the biological stress hormone), especially in those traumatized early in life (15), indicating developmental programming and vulnerability to psychopathology (13). In the presence of early life events, the cortisol metabolizing enzymes 5α-reductase and 11β-HSD type 2 activities are reduced (13). Yehuda and colleagues concluded that diminished cortisol metabolism could be a marker of primal susceptibility, potentially by attenuated peripheral catabolism of cortisol resulting in a reduction of the biological stress system sensitivity.

In SZ and BD, two studies by Steen and colleagues concluded with elevated cortisol metabolism (11, 16). Steen et al. reported elevated 5α- and 5β-reductase and 11β-HSD type 2 activity in SZ, whilst BD had intermediate levels between SZ and healthy controls (HC). However, overall 11β-HSD activity was not significantly altered. Elevated cortisol metabolism was proposed as a mechanism for HPA axis dysfunction in these disorders (11, 16, 17). However, the role of childhood trauma within this context is yet to be established. Stressful life events during childhood could lead to HPA axis dysregulation through altered systemic cortisol metabolism (18). Due to the unambiguous findings of the key enzymes 5α- and 5β-reductases in cortisol clearance, these two enzymes are of specific interest. They constitute the pathway with the most consistent finding of increased cortisol clearance in SZ and BD, and they are key enzymes in cortisol metabolism catalyzing irreversible conversion of cortisol and are both expressed in the liver. Moreover, cortisol has been shown to have an impact on cognition in in BD and SZ (1). However, it is yet unknown if poorer cognitive function evident in individuals with childhood trauma experiences (19, 20) associated with cortisol metabolism.

Our hypotheses are as follows: In patients with SZ and BD, childhood trauma and cognitive function will be related to cortisol metabolism activity, and cortisol metabolism will mediate the association between childhood trauma and cognitive function. We focused on the important rate-limiting enzymes 5α- and 5β-reductases as both consistently indicate increased hepatic cortisol clearance in SZ and BD.

## Methods

### Subjects

Participants were recruited consecutively from outpatient and inpatient units from four hospitals in Oslo as part of the larger NORMENT Research study. A sub-sample of subjects included from 2006 to 2010 had their urine sampled for estimation of systemic cortisol metabolizing activity. Enzyme activities were not estimated for individuals with diagnoses of hepatic- or renal disorder, thyroid dysfunction, or use of corticosteroid medications (11). The sub-sample included participants with information on urinary cortisol metabolites, childhood trauma variables from the CTQ screening interview and a standardized extensive cognitive battery, consisting of a total of 114 patients (63 schizophrenia spectrum disorder, SZ [34 schizophrenia; six schizophreniform disorder; nine schizoaffective disorder; 14 other psychosis]; 47 bipolar disorder, BD [32 bipolar I; six bipolar NOS; nine bipolar II]; and four major depressive disorder with psychotic features). The sample is part of larger sample of cortisol metabolism data previously reported on by Steen et al. (11, 16) the current sub-sample also had recording of childhood trauma variables.

Exclusion criteria for all groups were: poor fluency in Norwegian language or organic psychosis, or IQ below 70. The Regional Committee for Medical Research Ethics and the Norwegian Data Inspectorate approved the study. Written informed consent were given by all participants.

### Clinical Assessment

Clinical assessments were carried out by qualified clinical trained doctors, psychiatrists and clinical psychologists. The structured Clinical Interview for DSM-IV Axis I disorders (SCID-I) was used to assess diagnostic criteria’s of SZ and BD. Diagnostic reliability was found acceptable with overall agreement for DSM-IV diagnostic categories of 82% and the overall κ 0.77 (95% CI: 0.60–0.94). Positive and negative symptoms during the last seven days were assessed by the Positive and Negative Symptom Scale (PANSS) (21). Inter-rater reliability was satisfactory with intra-class correlation coefficients for PANSS subscales in the range of 0.71 to 0.73 (22). Function level was assessed using a split version of the Global Assessment of Functioning Scale [GAF; (23)].

### Cognitive Assessment

Cognitive assessment was performed by psychologists trained in standardized neuropsychological test batteries. The neuropsychological test battery was administered in a fixed order and took 3-hour with two breaks with refreshments. In this study, we specifically focused on measures sensitive to dysfunction in SZ and BD, and to stress (24–26). Five cognitive areas were assessed: 1) Verbal learning and memory; 2) Working memory 3) Executive function 4) Performance intelligence and 5) Verbal intelligence. Standardized z-scores were calculated based on healthy controls performance (mean and standard deviation) and collapsed into domain scores. The following neurocognitive domains were calculated:

*Learning and Memory* was assessed using the California Verbal Learning Test (CVLT) II, including sub items for learning, delayed recall and recognition (27). *Working memory* was assessed using Letter-Number Sequencing and Digit Span (WAIS-III) (28). **Ex***ecutive functioning* was assessed with the Verbal Fluency Test (Delis–Kaplan Executive Function Scale (D-KEFS) (27) with measures of phonetic fluency and semantic fluency. *Performance abilities* were measured using Block Design and Matrix Reasoning from the Wechsler Abbreviated Scale of Intelligence (WASI) (29). *Verbal abilities* were measured using Similarities and Vocabulary from WASI (29). All cognitive scores were presented as z-scores constructed based on the overall baseline mean and standard deviation from the healthy controls (healthy controls IQ score 113 ± 10.04; range 78–138) in the larger TOP sample [for more details see (30)].

### Childhood Trauma Questionnaire (CTQ)

The Childhood Trauma Questionnaire (CTQ) was applied to assess for traumatic events in childhood (see (30, 31), including emotional abuse (EA), physical abuse (PA), sexual abuse (SA), physical neglect (PN), and emotional neglect (EN). CTQ is a self-report questionnaire and each subscale is comprised of five items rated on a 5-point Likert scale ranging from 1 (never true) to 5 (very often true). For more detailed please see (30, 32).

### Design and Cortisol Measurements

All patients took part in the cognitive testing and routine blood withdrawal, and spot urine was sampled for analyses of urinary free cortisol (UFF), urinary free cortisone (UFE), allo-tetrahydrocortisol (aTHF), tetrahydrocortisol (THF) and tetrahydrocortisone (THE) (11). The Institute of internal medicine, University of Bergen and the Hormone Laboratory, Haukeland University Hospital performed the measurements of allo-THF, THF, THE, UFF and UFE based on liquid chromatography tandem mass spectrometry (LCMSMS) (for details see (17). Indexes of enzyme activities: Activities of the 5α- and 5β reductases were calculated with aTHF/UFF and THF/UFF indexes respectively (33) . Urinary creatinine was measured (Jaffé-reaction, Cobas Integra, Roche Diagnostics GmbH, Mannheim, Germany) to adjust for urine concentration.

### Statistical Analyses

Data were analyzed using IBM SPSS Statistics, Version 26.0. Spearman’s correlations were performed for the bivariate associations between childhood trauma, cortisol-metabolizing enzyme activities and cognitive domains. Independent sample t-tests were applied comparing enzyme activities of participants on regular treatment with at least one drug regularly of either antipsychotics, antidepressants or mood stabilizers to participants not on regular treatment with these pharmacological agents. Further analyses subdividing into number of drugs within each drug class were conducted using analysis of variance (ANOVA). Data were log transformed before analyzed by independent sample t-tests, ANOVA or multiple regression analyses. Hayes mediation model (34) was applied to investigate if cortisol metabolism mediated the relationship between a history of childhood trauma experiences and poorer cognitive functioning with built in bootstrapping. The mean and median time of the samples was 11 am. As no significant associations were observed for BMI levels, urine concentration and enzyme activities or (p >0.1), we did not adjust the results for BMI levels, or urine concentration. Regression models (including the mediation models) were adjusted for age, sex and diagnosis. Similar to (30), the four participants with MDD with psychotic features were added to the BD group to create an “affective group”. To rule out type 1 error, we adjusted for analyzing several cognitive domains and childhood trauma subtypes, applying a significance level of 0.01 instead of <0.05. As this was a hypothesis driven study, we decided this was appropriate without the possibility of losing vital information.

## Results

### Overview of the Sample

In the total sample, the mean age at inclusion was 32 years, and 60% were males. Mean years of education was thirteen. Patients with SZ were younger and had fewer years of education than patients with BD. Patients with SZ had more severe symptoms from the PANSS and poorer functioning from the GAF and scored poorer on all cognitive tests compared to the BD group. Patients with SZ also had higher 5α-reductase activity than BD, whilst contrary to the larger sample (11) no statistically significant association were observed between groups for 5β-reductases. There were no differences in childhood trauma experiences between SZ and BD (see **Table 1**). N = 82 (72%) of the patients were regularly prescribed antipsychotic medication, 43 (38%) were regularly receiving antidepressants and 31 (27%) were receiving anticonvulsants or lithium (mood stabilizers). No association was observed for enzyme activities comparing users and non-users of antipsychotics (independent sample t-test, 5α-reductase activity, t = 0.18, p = 0.86; 5β reductases, t = 0.32, p = 0.75), users and non-users of antidepressants (5α-reductase activity, t = −1.02, p = 0.31; 5β reductases, t = −0.83, p = 0.41), or of mood stabilizers (5α-reductase activity, t = −1.58, p = 0.12; 5β reductases, t = −0.21, p = 0.84). Further analysis within each drug class showed that 32 (28%) were not regularly prescribed antipsychotics, 67 (59%) were receiving one type of antipsychotic medication, and 15 (13%) were receiving at least two types of antipsychotics. No association was observed between the groups and cortisol metabolism (ANOVA, 5α-eductases: F = 0.25, p = 0.78; 5β-reductases, F = 0.53, p = 0.59). However, the current study is a subsample of Steen et al. (11) where analyses showed a nominal increase in 5β-reductase with use of antipsychotics. Seventy-one (62%) were not prescribed any regular antidepressants, 28 (25%) were receiving one type of antidepressant, nibe (8%) were receiving two types of antidepressants and six (5%) were receiving three or more types of antidepressants. No association was observed between groups and cortisol metabolism (ANOVA, 5α-eductases: F = 1.97, p = 0.12; 5β-reductases, F = 0.88, p = 0.45). As only one person was prescribed more than one type of mood stabilizer, only antipsychotics and antidepressants were broken down to number of different drugs.

**Table 1:** Demographics of the patients and clinical characteristics divided into schizophrenia and bipolar disorders.

	SZ N = 63	BD N = 51	Statistics
Age (mean ± SD)	28.9 ± 8.9	35.9 ± 12.2	F = 12.5, DF = 1, P = 0.001
Sex (M/F)	37/26	23/28	X^2^ = 2.1, DF = 1, P = 0.15
Years of education (mean ± SD)	12.2 ± 2.8	14.3 ± 3.6	F = 12.6, DF = 1, P = 0.001
GAF (mean ± SD)	44.3 ± 11.1	49.9 ± 10.9	F = 7.4, DF = 1, P = 0.007
PANSS total score	63.6 ± 20.1	45.7 ± 10.7	F = 32.7, DF = 1, P < 0.001
Childhood trauma total score (mean ± SD)	46.6 ± 15.9	45.1 ± 19.9	F = 0.28, DF = 1, P = 0.60
Physical Abuse, (mean ± SD)	7.6 ± 3.8	7.4 ± 4.4	F = 0.08, DF = 1, P = 0.79
Sexual Abuse (mean ± SD)	7.3 ± 4.4	7.0 ± 4.8	F = 0.15, DF = 1, P = 0.70
Emotional Abuse (mean ± SD)	11.1 ± 4.7	10.1 ± 5.4	F = 1.13, DF = 1, P = 0.29
Physical Neglect (mean ± SD)	12.8 ± 5.1	12.6 ± 5.4	F = 0.05, DF = 1, P = 0.83
Emotional Neglect (mean ± SD)	8.3 ± 3.1	7.5 ± 3.8	F = 1.22, DF = 1, P = 0.27
Memory (mean ± SD^)a^	−0.9 ± 1.4	−3.1 ± 1.2	F = 5.20, DF= 1, P = 0.02
Working memory (mean ± SD)^a^	−0.8 ± 0.8	−0.4 ± 0.9	F = 11.1, DF = 1, P = 0.001
Executive function (mean ± SD)^a^	−1.3 ± 1.1	−0.5 ± 1.2	F = 6.0, DF = 1, P = 0.02
Verbal abilities (mean ± SD)^a^	−1.3 ± 1.5	−0.6 ± 1.1	F = 7.9, DF = 1, P = 0.006
Perception and visuo-spatial abilities (mean ± SD)^a^	−1.1 ± 1.6	−0.6 ± 1.4	F = 3.3, DF = 1, P = 0.07
5β-reductase activity (mean ± SD)	96.9 ± 58.4	82.4 ± 56.3	F = 2.3, DF = 1, P = 0.13
5α-reductase activity (mean ± SD)	112.2 ± 103.3	75.3 ± 58.2	F = 6.3, DF = 1, P = 0.01

SZ, Schizophrenia; BD, bipolar disorder. 99.1% (n = 113) of the patients with estimated cortisol metabolism activity completed physical abuse subscale; 97.4% (n = 111) completed sexual abuse subscale; 98.2% (n = 112) completed emotional abuse subscale; 99.1% (n = 113) completed emotional neglect subscale, and 97.4% (n = 111) completed physical neglect subscale. 96.5% (n = 110) had complete data on years of education. a = z score.

### Cortisol Metabolism and Childhood Trauma

5β-reductase was positively correlated with physical abuse experiences (Spearman’s correlation [rho], r = 0.26; p = 0.005), moreover, correlations with sexual abuse (r = 0.20, p = 0.04), and emotional abuse (r = 0.17, p = 0.07) were indicated (see **Table 2**). 5α-reductase was positively correlated at trend level with emotional neglect (r = 0.18, p = 0.06). After correcting for confounders (age, sex and diagnosis) physical abuse was still nominally associated with higher 5β reductases (see **Table 3**).

**Table 2:** Association between cortisol metabolism, childhood trauma and cognitive function.

	5β reductases (n = 114)	5α Reductases (n = 114)
Physical abuse	r = 0.26 p = 0.005	r = 0.15 p = 0.10
Sexual abuse	r = 0.20 p = 0.035	r=0.12 p=0.22
Emotional abuse	r = 0.17 p = 0.07	r=0.12 p=0.19
Physical neglect	r = 0.07 p = 0.49	r = 0.06 p = 0.57
Emotional neglect	r = 0.12 p = 0.20	r = 0.18 p = 0.06
Memory	r = 0.05 p = 0.60	r = −0.02 p = 0.80
Working memory	r = −0.15 p = 0.11	r = −0.23 p = 0.01
Executive function	r = −0.15 p = 0.11	r = −0.19 p = 0.05
Verbal abilities	r = 0.04 p = 0.70	r = −0.10 p = 0.30
Perception and visuo-spatial abilities	r = −0.06 p = 0.53	r = −0.09 p = 0.34
GAF	r = −0.17 p = 0.08	r = −0.22 p = 0.02
PANSS	r = 0.14 p = 0.15	r = 0.23 p = 0.01

Spearman’s correlation; aTHF, allo-tetrahydrocortisol; THF, tetrahydrocortisol; THE, tetrahydrocortisone; UFF, urinary free cortisol; UFE, urinary free cortisone; 5α- and 5β reductases were calculated with aTHF/UFF and THF/UFF indexes respectively.

**Table 3:** Linear regression with possible confounders of the correlation between 5β reductases and physical abuse.

	B	SE	T	p value
Constant	4.05	0.26	15.47	˂0.001
Age	0.01	0.01	2.20	0.03
Sex	0.03	0.11	0.27	0.79
Diagnosis (SZ/BD)	−0.24	0.12	−2.05	0.04
Physical abuse	0.03	0.01	2.09	0.039

SZ, schizophrenia, BD, Bipolar disorders.

### Cortisol Metabolism, Cognitive Function and Clinical Characteristics

5α-reductases were negatively correlated with working memory (Spearman’s correlation [rho], r = −0.23, p = 0.01). An association with executive function (r = −0.19, p = 0.05) was also suggested. However, neither the association with working memory nor with executive function was statistically significant or at trend level after adjusting for age, sex and diagnosis (B = −0.13 SE = 0.09, t = −1.29, p = 0.21; B = −0.08, SE = 0.06, t = −0.75, p = 0.45, respectively). Similarly, 5α-reductases was positively correlated with total PANSS score (as a measure of more severe current symptoms, r = 0.23, p = 0.01) and negatively correlated at a nominal level with GAF-F (with lower GAF-F as a measure of poorer functioning, r = 0.22, p = 0.02). After adjusting for age, sex and diagnosis there were no longer a relationship between 5α-reductases and GAF-F or 5α-reductases and total PANSS score (B = −0.13, SE = 0.007, t = −1.34, p = 0.18; B = 0.08, SE = 0.004 t = 0.75, p = 0.45, respectively). 5β-reductase was not associated with the cognitive or clinical measures.

Lastly, *process* confirmed that cortisol metabolism (5α-reductase or 5β-reductase) did not mediate the association between childhood physical abuse and cognitive function (working memory or executive functioning, p >0.1; see adjusted values in **Supplementary Material Figures S1–4**). Based on the negative findings in **Table 2**, no other mediation analyses were ran.

## Discussion

Our study indicates that childhood adverse events are associated with elevated cortisol metabolism (5β-reductase) in adults with a SZ or BD disorder. After adjusting for potential confounders, this was only suggested for 5β-reductases and physical abuse. Cortisol metabolism may affect HPA axis activity (35), and is suggested as a factor for HPA axis dysfunction in SZ and BD (11, 16). Individuals with extreme stressful experiences in childhood, including PTSD have changes in their cortisol metabolism profile (13, 36). However prior to our study this had not yet been investigated in SZ or BD, even though patients with SZ and BD more often report childhood trauma experiences than individuals without a mental disorder (2).

Individuals with childhood trauma experiences have attenuated cortisol metabolism than those without trauma (13). Contrary to what observed in PTSD, a history of childhood trauma was accompanied by elevated cortisol metabolism in SZ and BD, specifically in individuals who also reported childhood physical abuse. These findings support a distinct cortisol metabolism profile in SZ and BD affected by childhood adverse experiences. Differences in cortisol metabolism between the diagnostic groups are supported by altered levels of cortisol in in PTSD compared to SZ or BD, with low cortisol levels reported in PTSD (13), while elevated diurnal cortisol levels are often reported in SZ or BD (9, 37, 38). Similarly, in major depression there are indications of reduced cortisol clearance (39, 40).

The suggested link between poorer cognitive function (working memory, executive functioning), and poorer clinical functioning (GAF and PANSS) and elevated cortisol metabolism (5α-reductases) was no longer statistically significant or at trend level after adjusting for potential confounders, such as age, sex and diagnosis. As discussed in Pruessner et al. (1), the HPA axis is vital for survival as it allows the organism to prepare for hostile events and to recover after stress experiences. On the other side, long-term activation of the biological stress system can have unfavorable effects on brain structure/function and behavior (41, 42). High levels of stress over time have been linked to changes in regional brain volumes and neuronal structure and function. In fact, severe and chronic stress, both in childhood and in adulthood, can cause neural cell death and atrophy of neuronal processes, and influence hippocampus neurogenesis and plasticity (43–45). Our negative findings suggest that sampling techniques focusing on long-term exposure of cortisol (such as cortisol in hair) may be a more robust marker to capture stress related brain changes within these groups than a one-time point sample targeting transitory cortisol levels, which can be affected by diurnal rhythm, activity levels and the day-night cycle (9).

This study had several strengths: The well-characterized large sample size with psychological assessment as well as biological markers (here 5α-reductase and 5β-reductase activity), and information on trauma history. Moreover, this study covers an important gap in the literature addressing cortisol metabolism and childhood trauma in SZ and BD. We focused our study on important rate limiting enzymes catalyzing irreversible conversions and showing consistent alterations in previous studies (11, 16).

Some limitations of the study should be mentioned: Firstly, within this cohort we did not have data on childhood trauma experiences for the healthy controls, thus we were unable to investigate the role of childhood trauma experiences on cortisol metabolism in a healthy population. However numerous of studies show that patients with SZ and BD report more childhood trauma experiences than HC (1, 2, 46), suggesting vulnerability for alteration in cortisol metabolism in patients with SZ or BD due to higher trauma exposure. Furthermore, information about childhood trauma was assessed in adults using the Childhood Trauma Questionnaire (CTQ), a retrospective questionnaire about trauma experiences in childhood. A recent meta-analysis study found low overlap between retrospective and prospective data on childhood trauma (47), however large heterogeneity was reported across studies. Encouragingly, convergent validity of prospective and retrospective data has been suggested as they are associated with similar outcomes (48), supporting the use of retreospective design. Although our finding of an association between higher cortisol metabolism and reports of physical abuse were independent of age, sex, and diagnoses, we cannot rule out that other confounding factors may be present that we have not adjusted for. Although we adjusted for number of trauma subtypes and number of cognitive tests using a more stringent P value of 0.01 than the standard of less than 0.05, we cannot rule out potential spurious findings. Lastly, after adjusting for potential confounders we no longer found an association between cortisol metabolism and cognitive (working memory, executive functioning) or clinical features (GAF, PANSS). However, we cannot rule out the presence of subgroups (clusters) within the sample. For example, it could be subgroups of individuals characterized by high levels of trauma, elevated cortisol metabolism and poorer cognitive function, and other clusters characterized by high load of genetic vulnerability, poor cognition and no history of childhood trauma. Due to our relatively small sample (n = 114) we were not able to perform these additional cluster analyses.

To conclude, our study indicates that physical abuse in childhood is associated with elevated cortisol metabolism (5β-reductase) in adults with a SZ or BD disorder, demonstrating a distinct cortisol metabolism profile in SZ and BD affected by childhood adverse experiences. Whether cortisol metabolism is associated with cognitive and clinical characteristics within subgroups of these disorders needs to be further investigated for example by applying cluster analysis in larger samples.

## Data Availability Statement

The datasets generated for this study are available on request to the corresponding author.

## Ethics Statement

The studies involving human participants were reviewed and approved by Regional Committee for Medical Research Ethics and the Norwegian Data Inspectorate. The patients/participants provided their written informed consent to participate in this study.

## Author Contributions

All authors (MA, TU, AI, IM, OA, NS) listed have made substantial, direct, and intellectual contribution to the work and approved it for publication.

## Funding

We thank the patients who took part in the study and the TOP study group researchers who contributed to the data collection. This study was funded by grants from the South-Eastern Norway Health Authority (#2017060) and the Narsad Young Investigator Award to MA (#22388). The study was also funded by the Research Council of Norway (#22327), the KG Jebsen Stiftelsen.

## Conflict of Interest

The authors declare that the research was conducted in the absence of any commercial or financial relationships that could be construed as a potential conflict of interest.
