# Supplementary material for: Childhood Trauma Is Nominally Associated With Elevated Cortisol Metabolism in Severe Mental Disorder
Source: Front Psychiatry. 2020 May 14;11:391. doi: 10.3389/fpsyt.2020.00391 (PMC7247816; doi:10.3389/fpsyt.2020.00391)
Supplement: Supplementary file 1 [file DataSheet_1.docx]

**SUPPLEMENTARY MATERIAL**

**Figure S1**. 5α-reductase and working memory


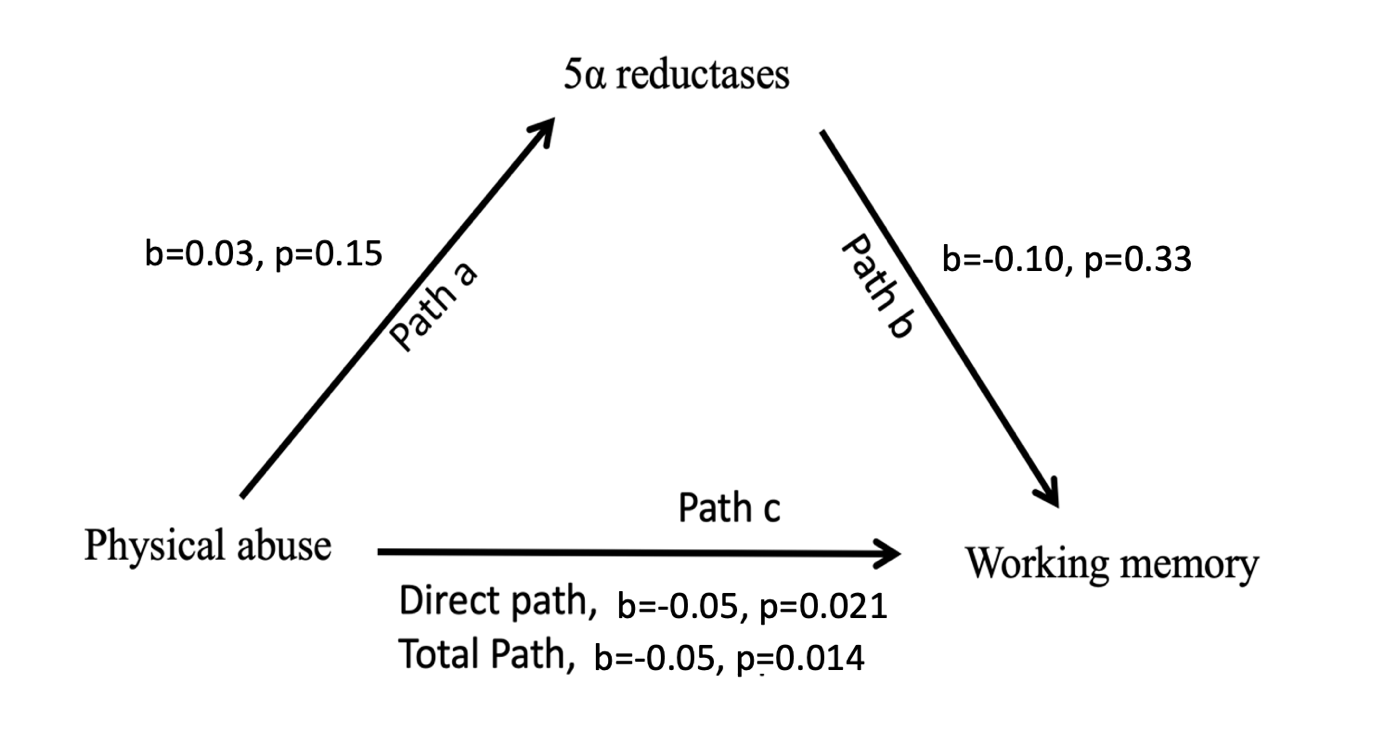


Hayes, mediation model. Adjusted for age, sex and diagnosis (schizophrenia, bipolar disorder).

**Figure S2.** 5α-reductase and executive functioning


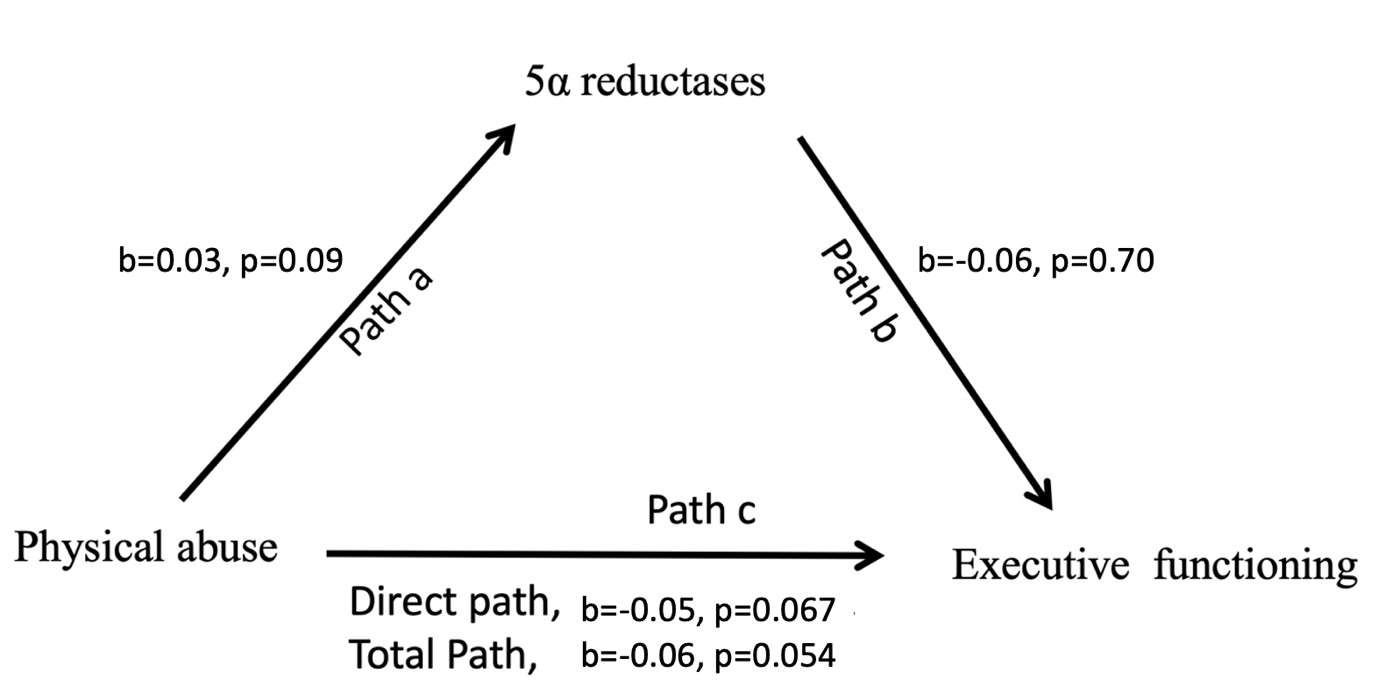


Hayes, mediation model. Adjusted for age, sex and diagnosis (schizophrenia, bipolar disorder).

**Figure S3**. 5β-reductase and working memory


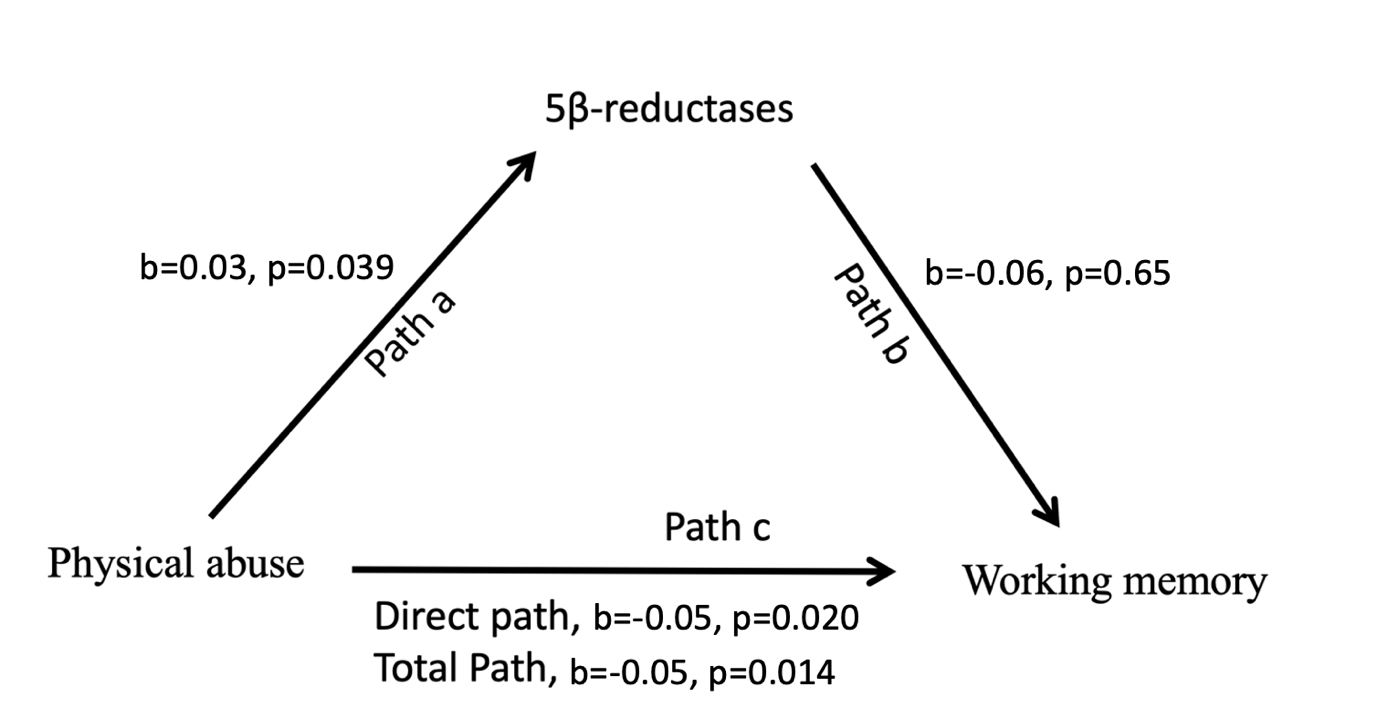


Hayes, mediation model. Adjusted for age, sex and diagnosis (schizophrenia, bipolar disorder).

**Figure S4.** 5β-reductase and executive functioning


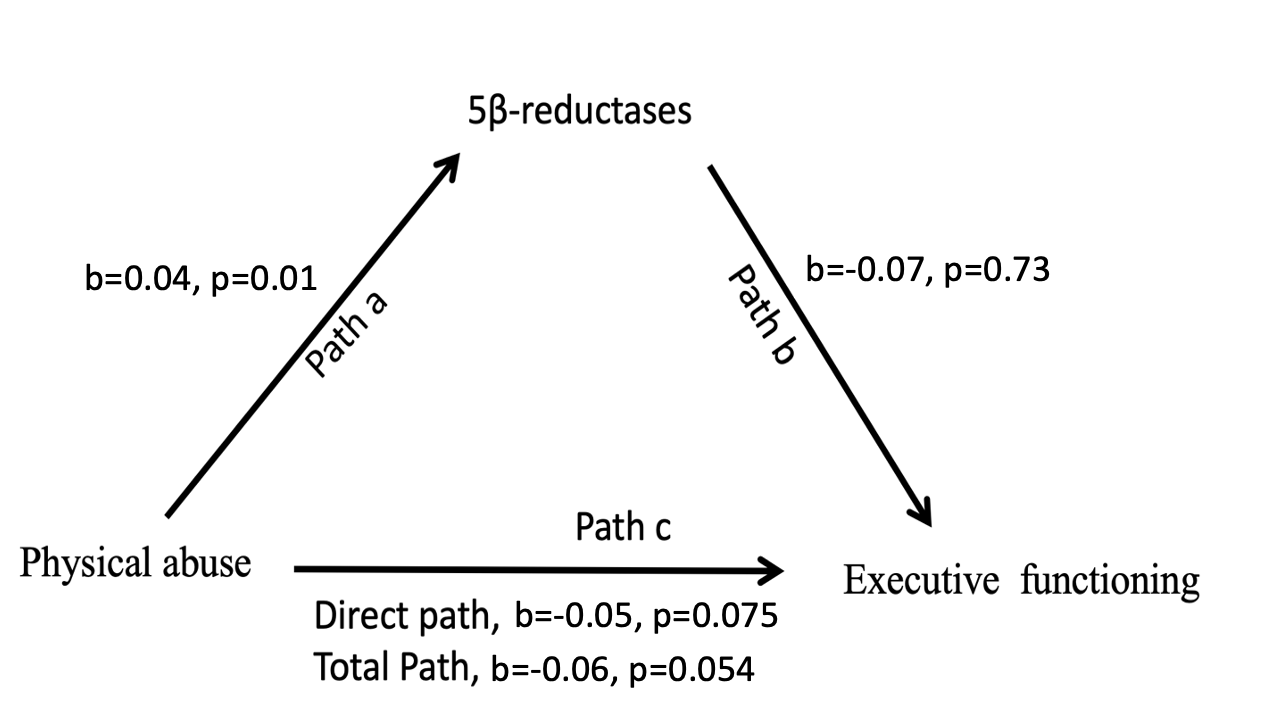


Hayes, mediation model. Adjusted for age, sex and diagnosis (schizophrenia, bipolar disorder).
